# Anatomical and Functional Outcomes of Internal Limiting Membrane Flap-Assisted Removal of Retained Subretinal Perfluorocarbon Liquid: A Case Series of Three Patients

**DOI:** 10.3390/jcm15218344

**Published:** 2026-10-26

**Authors:** Sławomir Cisiecki, Nela Poznańska, Joanna Bogatko, Karolina Bonińska, Maciej Bednarski

**Affiliations:** Department of Ophthalmology, Karol Jonscher Municipal Medical Centre, Milionowa 14, 93-113 Łódź, Poland

**Keywords:** internal limiting membrane, pars plana vitrectomy, perfluorocarbon liquid, PFCL removal, retinal detachment, subretinal PFCL

## Abstract

**Background**: Retained subretinal perfluorocarbon liquid (PFCL) is a potentially vision-threatening complication of retinal detachment surgery. No standardized surgical approach exists. This study describes an internal limiting membrane (ILM) flap-assisted removal technique. **Methods**: Three symptomatic patients underwent 23-gauge pars plana vitrectomy for retained subretinal PFCL, subfoveal in one eye and parafoveal in two. A temporally hinged ILM flap was reflected and stabilized with an intravitreal PFCL bubble, and 39-gauge cannulas were used for focal transretinal puncture and subsequent aspiration of the retained PFCL. The flap was repositioned using a new PFCL bubble, followed by fluid–air exchange. Outcomes were assessed using optical coherence tomography (OCT), best-corrected visual acuity (BCVA), and patient-reported visual symptoms over 8–33 months. **Results**: Complete PFCL removal was achieved in all cases. Final BCVA was better than immediately before PFCL removal in all eyes. OCT demonstrated variable structural recovery, with varying degrees of residual structural abnormalities. No epiretinal membrane (ERM) formation was detected during follow-up. **Conclusions**: The technique enabled complete PFCL removal in three selected patients. Given the small sample, case heterogeneity, and potential confounding effects of previous retinal detachment and concomitant procedures, the findings support technical feasibility but do not establish efficacy, safety, or superiority over existing approaches.

## 1. Introduction

Retained subretinal perfluorocarbon liquid (PFCL) following vitrectomy for retinal detachment is a potentially serious complication that can lead to permanent visual impairment. Its incidence is estimated at 1–11% [1,2]. Risk factors include retinal breaks located posterior to the equator, large retinal tears, and vitrectomy procedures requiring extensive retinotomies, particularly those involving 360° [1,2]. Subretinal PFCL may lead to structural damage involving the retinal pigment epithelium (RPE) and photoreceptors, resulting in thinning of the outer retinal layers and localized scotomas corresponding to the affected retinal area [3,4]. Surgical removal of subretinal PFCL should be considered when the fovea is involved or when there is a risk of migration to the fovea, as it may permit partial recovery of retinal function [3,4].

Several surgical approaches have been described, including direct aspiration of subretinal PFCL using small-gauge cannulas [5,6], removal through a therapeutic macular hole [7], displacement techniques involving subretinal balanced salt solution (BSS) injection [8,9], and internal limiting membrane (ILM) flap repositioning combined with PFCL aspiration [10]. However, no standardized surgical approach has been established. In this case series, we describe an ILM flap-assisted technique for the management of retained subretinal PFCL and evaluate its anatomical and functional outcomes.

## 2. Materials and Methods

### 2.1. Study Design

This retrospective case series included three patients treated at a single tertiary ophthalmology center and was based exclusively on anonymized pre-existing medical records and procedures performed for clinical indications. Written informed consent was obtained from all three patients for the use of their anonymized clinical data in retrospective research. The study was conducted in accordance with the Declaration of Helsinki.

### 2.2. Patients

Medical records covering the period from July 2023 through August 2026 were retrospectively reviewed. All three patients who met the eligibility criteria were included consecutively, with no eligible cases excluded.

The eligibility criteria were as follows: (1) subfoveal or parafoveal PFCL confirmed by optical coherence tomography (OCT) following pars plana vitrectomy for rhegmatogenous retinal detachment; (2) visual symptoms, including reduced visual acuity and/or metamorphopsia; and (3) sufficient ILM tissue for flap creation. Accordingly, asymptomatic patients and those in whom previous ILM peeling precluded creation of the required flap were not eligible. As systematic screening for retained PFCL was not performed, the number of asymptomatic cases during the study period could not be determined.

The primary retinal detachment repair had been performed at our center in two patients and at another institution in one patient. All subsequent subretinal PFCL-removal procedures were performed at our tertiary referral center by the same vitreoretinal surgeon (S.C). The retained PFCL was subfoveal in one case and parafoveal in two.

### 2.3. Surgical Procedure

In all cases, three-port 23-gauge pars plana vitrectomy was performed under retrobulbar anesthesia. The ILM was stained for 10 s with 0.05% indocyanine green (ICG; 0.5 mg/mL; Verdye^®^, Diagnostic Green GmbH, Aschheim-Dornach, Germany) diluted in BSS [11].

ILM peeling was initiated nasal to the fovea. A tongue-shaped flap approximately two optic disc diameters in size was created within the vascular arcades, preserving a temporal hinge. The flap was reflected temporally and stabilized with approximately 1 mL of PFCL (ARCALINE^®^, Arcadophta, Toulouse, France) placed over the exposed retina.

A focal transretinal puncture was created at the top of the retained PFCL droplet using a 39-gauge cannula (DORC, Zuidland, The Netherlands). A separate 39-gauge aspiration cannula, connected through silicone tubing to a 1 mL insulin syringe, was then used to aspirate the subretinal PFCL. Manual suction was applied by the scrub nurse under the surgeon’s guidance. Complete removal was assessed using intraoperative OCT. The retained PFCL appeared as a well-circumscribed, rounded subretinal cavity. Removal was considered complete when this characteristic rounded configuration was no longer visible and was replaced by a flattened hyporeflective defect within the retinal layers, resembling the OCT appearance of flat-open macular hole closure (Supplementary Video S1). Postoperative OCT confirmed the absence of residual PFCL in all cases.

The stabilizing PFCL bubble was then aspirated. A new PFCL bubble was introduced temporally and gradually enlarged to reposition the flap without inversion. The PFCL was removed during fluid–air exchange. Case-specific modifications and final tamponade are described in the individual case descriptions. Patients were instructed to maintain face-down positioning for 24 h. The principal surgical steps are outlined in the schematic representation (Figure 1) and Supplementary Video S1. The schematic illustration of the surgical technique (Figure 1) was generated using an AI tool based on detailed instructions provided by the authors. The illustration was reviewed and verified by the authors.

### 2.4. Outcome Measures

Anatomical outcomes were assessed using OCT to confirm PFCL removal and evaluate postoperative retinal structural changes. Macular OCT examinations were performed using Spectralis (Heidelberg Engineering, Heidelberg, Germany) and Topcon OCT devices (Topcon Corporation, Tokyo, Japan). Scans covered the macular region, with the same retinal areas examined during follow-up. OCT images were qualitatively assessed for retinal layer continuity and structural abnormalities. PFCL droplet height was measured on OCT as the maximum vertical distance from the apex of the droplet to its base at the level of the RPE. Functional outcomes were assessed using best-corrected visual acuity (BCVA) and patient-reported visual symptoms, including metamorphopsia and scotomas, recorded during routine follow-up. No standardized questionnaires were used to assess these symptoms. BCVA was measured and recorded on the logarithm of the minimum angle of resolution (logMAR) scale whenever chart-based acuity testing was possible. Counting-fingers and hand-motion measurements were converted to approximate logMAR equivalents for descriptive purposes. Counting fingers at a specified distance was converted using the method described by Karanjia et al., whereas hand motion and counting fingers without a specified distance were converted according to Schulze-Bonsel et al. [12,13]. These conversions were considered semiquantitative estimates and were not treated as equivalent to standardized chart-based logMAR measurements. The maximum follow-up duration was 33 months.

## 3. Results

### 3.1. Case 1

A 54-year-old male presented to our clinic with sudden deterioration of visual acuity in the right eye. Fundus examination revealed extensive retinal detachment involving all quadrants, with the macula detached. A 23-gauge phacovitrectomy with implantation of a bag-in-the-lens (BIL) intraocular lens (IOL; BIL, Tassignon; Morcher GmbH, Stuttgart, Germany) was performed, along with removal of vitreoretinal traction, extensive 360° endolaser photocoagulation, including treatment around the retinal breaks, and 12% C_2_F_6_ gas tamponade. At the 1-month follow-up, fundus examination and OCT revealed a parafoveal PFCL droplet with a maximum vertical height of 634 μm, situated superior to the foveal center (Figure 2A). The symptomatic location of the droplet was considered an indication for surgical removal. At the examination immediately preceding reoperation, BCVA in the right eye was 1.0 logMAR, and the patient reported significant metamorphopsia. Repeat vitrectomy was performed using the ILM flap-assisted technique and air tamponade.

#### Postoperative Course

On the first postoperative day, the patient required an additional intervention consisting of iris repositioning due to iris capture. At the 10-day postoperative visit, BCVA in the operated eye was limited to counting fingers (≈1.9 logMAR), and OCT demonstrated no residual PFCL (Figure 2B). At the 7-week follow-up, visual acuity improved to 0.6 logMAR, with patient-reported resolution of metamorphopsia. A dilated tonic pupil was observed in the operated eye, which may have affected visual function.

At the final follow-up, 9 months after PFCL removal, BCVA was 0.4 logMAR. OCT confirmed the absence of residual PFCL and demonstrated localized disorganization of the retinal layers with a focal RPE defect at the previous PFCL location, similar to the early postoperative findings (Figure 3).

### 3.2. Case 2

A 35-year-old male presented to our clinic with decreased visual acuity in the right eye after retinal detachment surgery at another center. The primary procedure, performed 1 month earlier, included vitrectomy with Densiron^®^ silicone oil tamponade (Fluoron GmbH, Ulm, Germany). The patient’s ocular history included laser-assisted in situ keratomileusis (LASIK) in both eyes 6 years previously. At presentation, fundus examination and OCT revealed a subfoveal PFCL droplet with a maximum vertical height of 316 μm (Figure 4A), with BCVA of 1.7 logMAR. Approximately 6 weeks after the primary surgery, repeat phacovitrectomy for subretinal PFCL removal was performed. The procedure included phacoemulsification with BIL IOL implantation, silicone oil removal, vitrectomy with endolaser photocoagulation, subfoveal PFCL extraction using the ILM flap-assisted technique, and 12% C_2_F_6_ gas tamponade. The gas tamponade was selected based on the surgeon’s individualized clinical assessment, considering the patient’s young age as a potential risk factor for proliferative vitreoretinopathy (PVR) [14], with the aim of providing prolonged retinal support. These concomitant procedures represent important potential confounders of the postoperative visual outcome.

#### Postoperative Course

At the 4-week follow-up, BCVA was limited to hand motion (≈2.3 logMAR), and OCT revealed an outer retinal defect at the fovea (Figure 4B). OCT examinations performed at subsequent follow-up visits demonstrated progressive restoration of the outer retinal layers and foveal architecture, accompanied by a gradual improvement in visual acuity (Figure 5). At the final follow-up, 8 months after PFCL removal, BCVA was 0.05 logMAR, with substantial restoration of the outer retinal layers and foveal contour, although residual focal abnormalities persisted (Figure 6).

### 3.3. Case 3

A 50-year-old male presented to our clinic with sudden visual deterioration in the left eye. Visual acuity was reduced to hand motion (≈2.3 logMAR). Fundus examination revealed retinal detachment involving all four quadrants, with the macula detached, and a full-thickness macular hole (Figure 7). Five days after the initial examination, the patient underwent 23-gauge phacovitrectomy with BIL IOL implantation and 12% C_2_F_6_ gas tamponade. An inverted ILM flap was used to close the macular hole, and endolaser photocoagulation was applied around the retinal breaks. On the first postoperative day, the retina was attached, with approximately three-quarters of the vitreous cavity filled with gas.

At the 3-week follow-up, the retina remained attached, and BCVA was 1.0 logMAR. OCT revealed a parafoveal PFCL droplet with a maximum vertical height of 930 μm (Figure 8A). By the day of reoperation, BCVA had deteriorated to counting fingers at 3 m (≈1.3 logMAR). Six weeks after the primary surgery, repeat vitrectomy for subretinal PFCL removal was performed.

During reoperation, the residual temporal ILM was mobilized while preserving its temporal attachment and elevated together with the pre-existing inverted ILM flap, thereby forming a composite hinged–inverted flap complex. The reflected complex was stabilized with an intravitreal PFCL bubble placed over the exposed retina. After aspiration of the retained subretinal PFCL, the stabilizing bubble was removed. A new intravitreal PFCL bubble was then introduced temporally and gradually enlarged to reappose the composite flap complex over the macula. After reapposition, the pre-existing component remained inverted, whereas the newly mobilized hinged component retained its native orientation. The PFCL was removed during fluid–air exchange, leaving an air tamponade.

#### Postoperative Course

On the first postoperative day, BCVA was limited to hand motion (≈2.3 logMAR). At the 1-week follow-up, BCVA was 1.4 logMAR, and OCT confirmed the absence of residual subretinal PFCL (Figure 8B). During subsequent follow-up visits, BCVA fluctuated between 1.0 and 1.6 logMAR, with values remaining close to 1.0 logMAR during the later postoperative period.

At the final follow-up, 33 months after PFCL removal, BCVA was 1.0 logMAR. The final OCT confirmed the absence of residual PFCL and preserved overall retinal layer continuity, although focal architectural disorganization, retinal thickening, and a localized RPE defect persisted at the previous PFCL location (Figure 9).

### 3.4. Overall Outcomes

The procedure enabled complete removal of retained subretinal PFCL in all three selected cases, as confirmed by postoperative OCT. Despite prior vitrectomy in all three eyes, ILM peeling and flap mobilization were performed without technical difficulties. Structural outcomes were variable. In Case 2, serial OCT demonstrated progressive improvement in outer retinal layer continuity and foveal contour, with residual structural abnormalities persisting at the final follow-up. In Cases 1 and 3, overall retinal continuity was preserved; however, localized abnormalities persisted in the retinal areas where the subretinal PFCL droplets had previously been located, including retinal layer disorganization, focal thickening, outer retinal abnormalities, and localized RPE defects.

At the final follow-up, BCVA was better than immediately before PFCL removal in all three cases, changing from 1.0 to 0.4 logMAR in Case 1, from 1.7 to 0.05 logMAR in Case 2, and from counting fingers at 3 m (≈1.3 logMAR) to 1.0 logMAR in Case 3. However, early postoperative deterioration and subsequent fluctuations were observed. The observed improvements in BCVA should be interpreted in the context of transient iris capture and a tonic pupil in Case 1, concomitant cataract surgery and silicone oil removal in Case 2, and previous macula-off retinal detachment and a full-thickness macular hole in Case 3, with additional uncertainty from the approximate conversion of counting-fingers acuity to logMAR.

Follow-up ranged from 8 to 33 months. No epiretinal membrane (ERM) formation was detected during the available follow-up; however, this represents a descriptive observation and does not permit conclusions regarding ERM risk. Metamorphopsia resolved in Case 1, and no patient spontaneously reported persistent metamorphopsia or scotomas. No standardized questionnaire, Amsler grid, microperimetry, or formal visual-field testing was used to assess these symptoms. The clinical course and visual outcomes of all three patients are summarized in Table 1.

## 4. Discussion

The surgical management of retained subretinal PFCL remains challenging, and the described techniques can be broadly classified as direct aspiration or displacement of the PFCL droplet. Direct approaches include aspiration through a focal retinal puncture using small-gauge cannulas or microneedles [5], ILM peeling followed by aspiration with a flexible 38-gauge cannula [6], and removal through a therapeutically created macular hole followed by gas tamponade [7]. Displacement techniques involve inducing a localized retinal detachment by subretinal BSS injection, followed by fluid–air exchange and postoperative upright positioning to move the PFCL toward the peripheral retina [8,9]. These methods differ in their retinal entry sites, use of intentional retinal detachment, and management of the ILM.

The approach most closely related to ours was described by Chen et al. [10]. Both techniques involve temporary reflection of an ILM flap, aspiration of the retained PFCL, and subsequent flap repositioning. Chen et al. created a tongue-shaped ILM flap, made a separate microretinal incision with a microvitreoretinal blade, aspirated the PFCL with a flute needle, and repositioned the flap with forceps. In our technique, the reflected flap was stabilized with an intravitreal PFCL bubble. A 39-gauge cannula was used to create a focal transretinal puncture at the top of the retained droplet, and a separate 39-gauge aspiration cannula was used to aspirate the PFCL. This approach avoids the need for a separate blade-created incision. Compared with the technique described by Chen et al., our use of an intravitreal PFCL bubble to stabilize the reflected ILM flap during aspiration, followed by a second PFCL bubble to facilitate flap reapposition, may limit unintended flap movement and reduce the risk of accidental flap aspiration or mechanical damage. In contrast to displacement techniques, our approach does not require intentional BSS-induced macular detachment. Whether these differences reduce retinal trauma or improve clinical outcomes requires comparative evaluation.

In our technique, the 39-gauge cannula creates a focal transretinal puncture that is expected to self-seal. If the entry site enlarges during aspiration, repositioning the ILM flap may provide an additional tissue layer over the resulting defect. The preserved ILM may also serve as a biological scaffold supporting cellular migration and tissue remodeling. Studies of inverted ILM flaps in macular hole surgery have shown that ILM components, including type IV collagen, fibronectin, and laminin, can support Müller cell proliferation and migration. Activated Müller cells may also produce neurotrophic factors and basic fibroblast growth factor involved in tissue repair [15,16,17]. Similar processes could theoretically promote glial bridging, closure of the focal retinal defect, and subsequent retinal remodeling after PFCL removal.

An additional potential advantage is the preservation of the native orientation of the hinged ILM flap. Maintaining its temporal attachment provides a reference axis that allows the flap to be returned to its original position without inversion or rotation. This applied to the entire hinged flap in Cases 1 and 2 and to the newly mobilized hinged component in Case 3; the pre-existing inverted component in Case 3 remained inverted. Repositioning the hinged flap in its native orientation keeps its retinal-facing surface apposed to the retina. Proliferating glial cells may therefore remain along the retinal surface rather than being transposed into the retinal defect. This could reduce the likelihood of glial plug formation reported after inverted ILM flap surgery [18] and support surface remodeling without excessive glial proliferation within the defect. The potential role of these cellular mechanisms in retinal repair following PFCL removal remains hypothetical.

Several case-specific factors complicate the interpretation of the functional outcomes. In Case 1, transient iris capture required iris repositioning and was considered a potential confounder, although its contribution to the long-term visual outcome remains uncertain. Its occurrence should also be interpreted in the context of the BIL design. Although this design limits contact between the posterior iris and the anterior capsule, thereby reducing posterior synechiae [19], its haptic configuration may allow the iris to pass beneath a haptic after pupillary dilation, resulting in iris capture [20]. A tonic pupil was also observed, possibly related to short ciliary nerve injury following extensive 360° endolaser photocoagulation, and may have affected visual function [21]. In Case 2, PFCL removal was combined with silicone oil removal, phacoemulsification, BIL IOL implantation, additional endolaser treatment, and 12% C_2_F_6_ gas tamponade. In Case 3, the previous macula-off retinal detachment and full-thickness macular hole treated with an inverted ILM flap may have limited the potential for visual recovery.

Consequently, the respective contributions of PFCL removal and ILM flap repositioning cannot be separated from the effects of previous retinal detachment and concomitant procedures, and the functional outcomes should be interpreted as descriptive observations only. The size of the retained PFCL droplets and the duration of their subretinal presence may also have influenced visual recovery [22].

This study is limited by its retrospective design, small sample size, absence of a control group, and heterogeneity in PFCL location, tamponade, and follow-up duration. These limitations prevent conclusions regarding efficacy, safety, or superiority over existing techniques.

### Future Directions

Future studies should evaluate this technique in larger, multicenter cohorts using standardized OCT imaging and visual acuity measurements. Metamorphopsia and scotomas should be assessed using standardized methods, such as Amsler-grid testing, microperimetry, formal visual-field testing, and validated questionnaires, rather than relying solely on spontaneous patient reports. Comparative studies are needed to assess technical success, reproducibility, anatomical and functional outcomes, and adverse events relative to alternative approaches for subretinal PFCL removal. Longer follow-up should evaluate retinal entry-site closure, flap stability, retinal microstructural remodeling, glial proliferation, and ERM formation.

## 5. Conclusions

In this retrospective series, the described ILM flap-assisted technique enabled complete removal of retained subretinal PFCL in all three selected symptomatic patients. Structural outcomes were variable, and final BCVA was better than immediately before PFCL removal in all three cases, although early postoperative deterioration and subsequent fluctuations occurred during follow-up. The described technique may therefore represent an additional surgical option for consideration in selected cases of retained subretinal PFCL. However, larger controlled studies are required to assess the safety and reproducibility of the technique and to determine its potential anatomical and functional benefits.

## Figures and Tables

**Figure 1:**
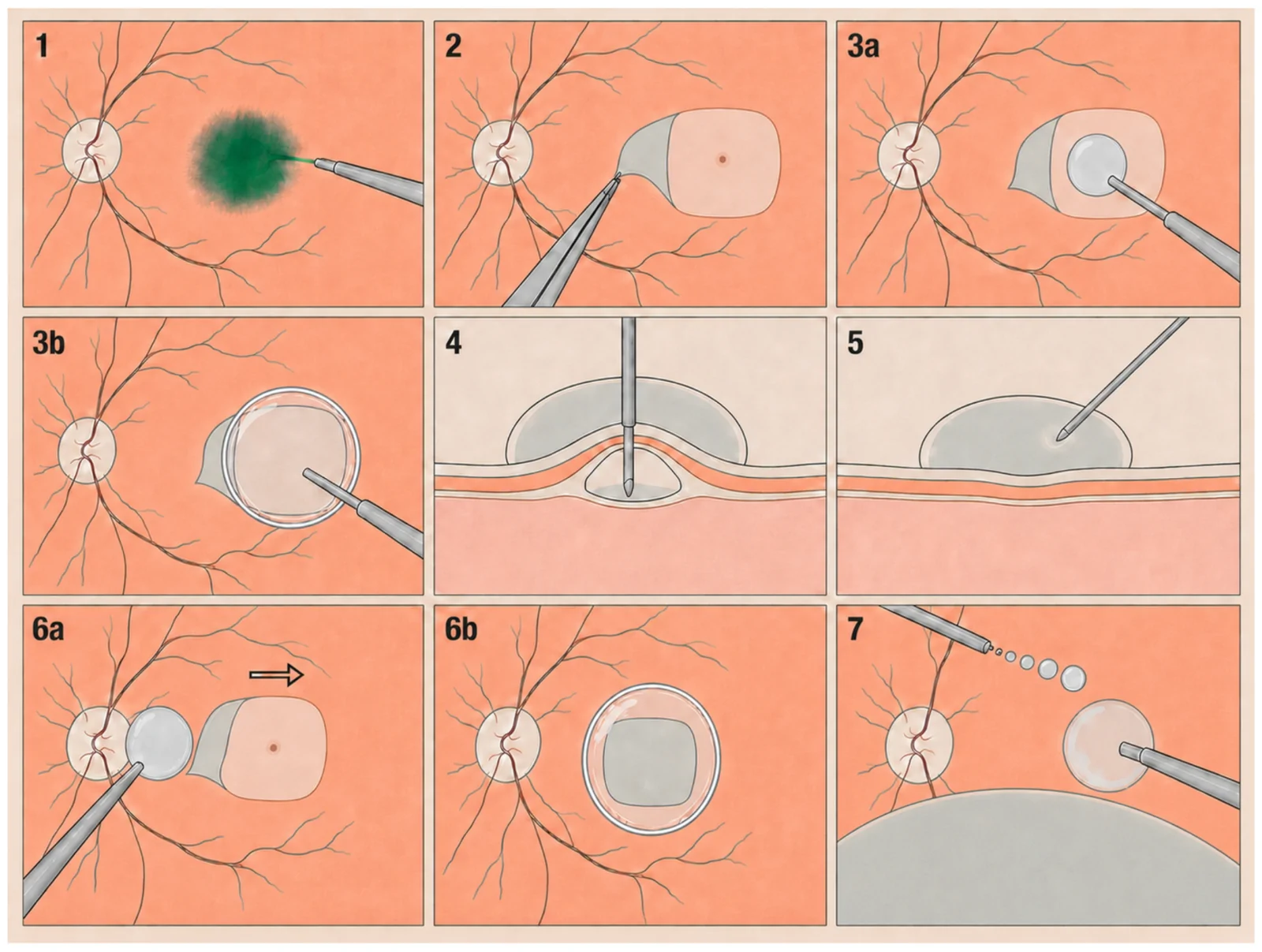
Schematic representation of the ILM flap-assisted technique for removal of retained subretinal PFCL. (**1**) Indocyanine green staining of the ILM. (**2**) Creation of a tongue-shaped ILM flap with preservation of a temporal hinge. (**3a**,**3b**) Reflection and stabilization of the flap with an intravitreal PFCL bubble placed over the exposed retina. (**4**) Creation of a focal transretinal puncture at the top of the retained PFCL droplet using a 39-gauge cannula, followed by aspiration of the subretinal PFCL through the same puncture. (**5**) Aspiration of the stabilizing intravitreal PFCL bubble. (**6a**,**6b**) Temporal introduction and gradual enlargement of a new intravitreal PFCL bubble adjacent to the reflected flap to facilitate flap reapposition without inversion. (**7**) Removal of the intravitreal PFCL during fluid–air exchange. The schematic illustrates the general sequence used in all three cases. In Case 3, the residual temporal ILM was mobilized together with the pre-existing inverted ILM flap to form a composite hinged–inverted flap complex.

**Figure 2:**
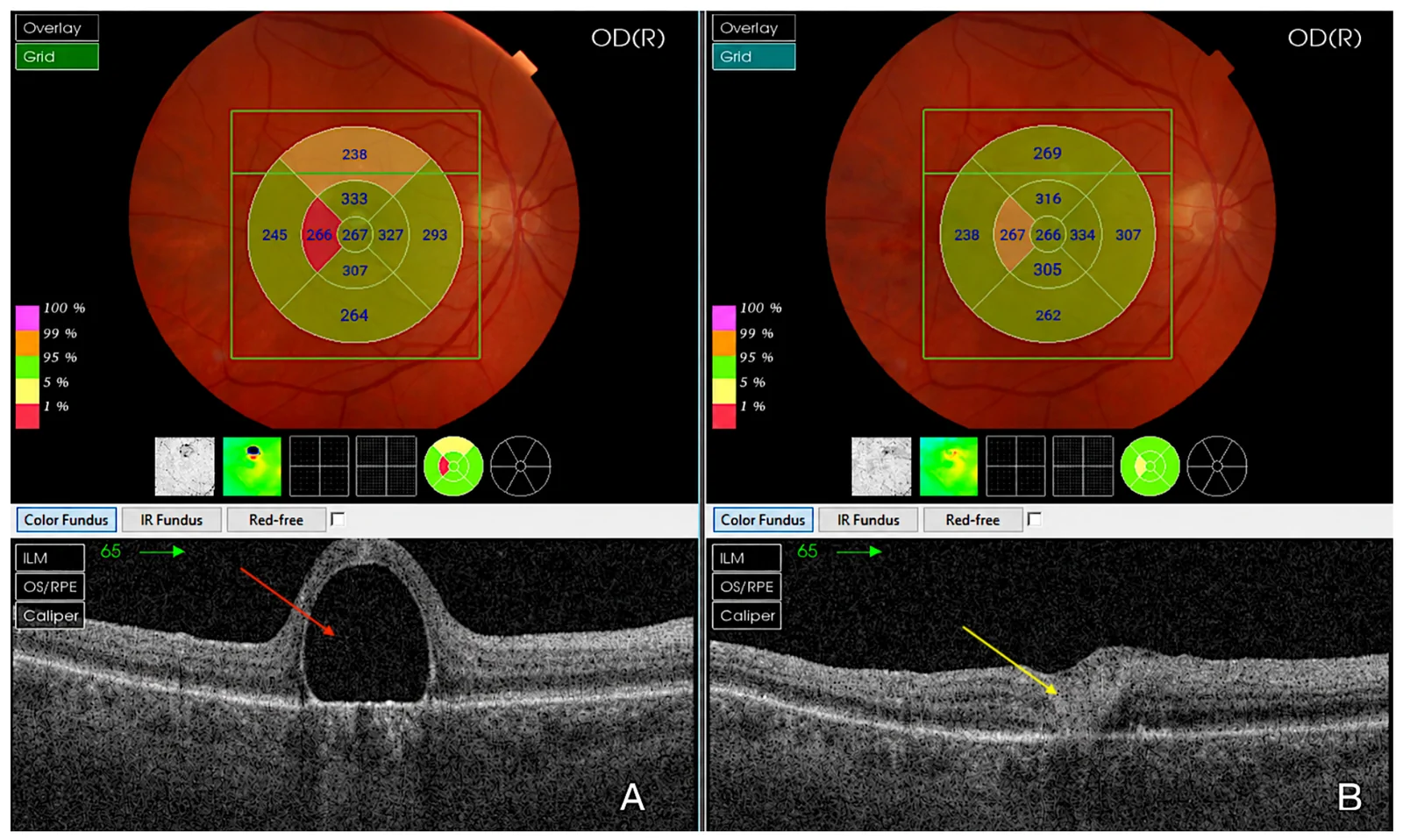
Case 1. (**A**) Preoperative OCT showing a parafoveal PFCL droplet (red arrow), with a maximum vertical height of 634 μm, located superior to the foveal center and overlying a focal RPE defect; continuity of the overlying retinal layers is preserved. (**B**) OCT at the 10-day follow-up showing complete removal of the PFCL and preserved overall retinal layer continuity, with focal architectural disorganization and a localized RPE defect at the previous PFCL location (yellow arrow).

**Figure 3:**
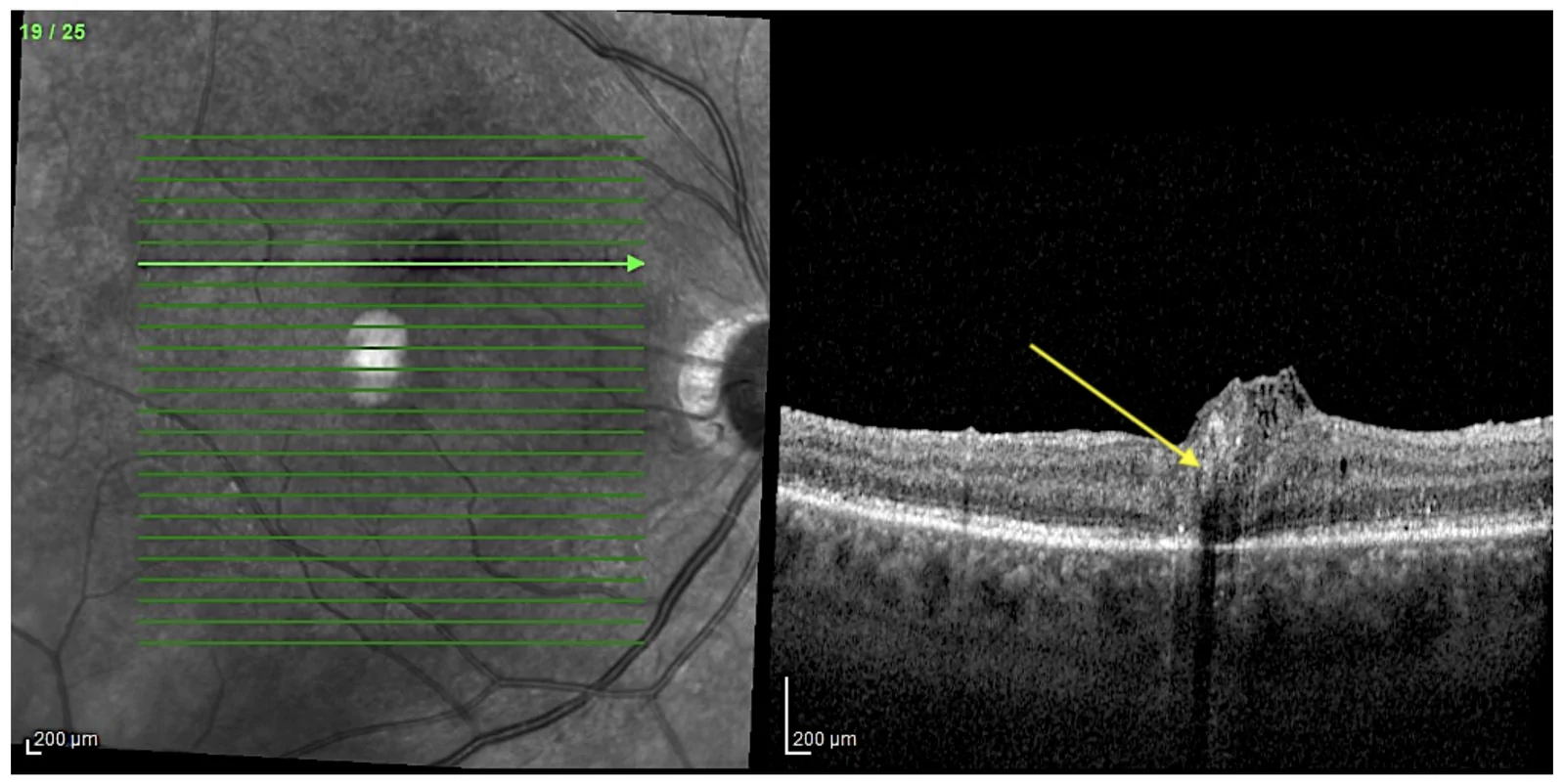
Case 1. Final OCT at the 9-month follow-up showing no residual PFCL, localized retinal layer disorganization, and a focal RPE defect at the previous PFCL location (yellow arrow).

**Figure 4:**
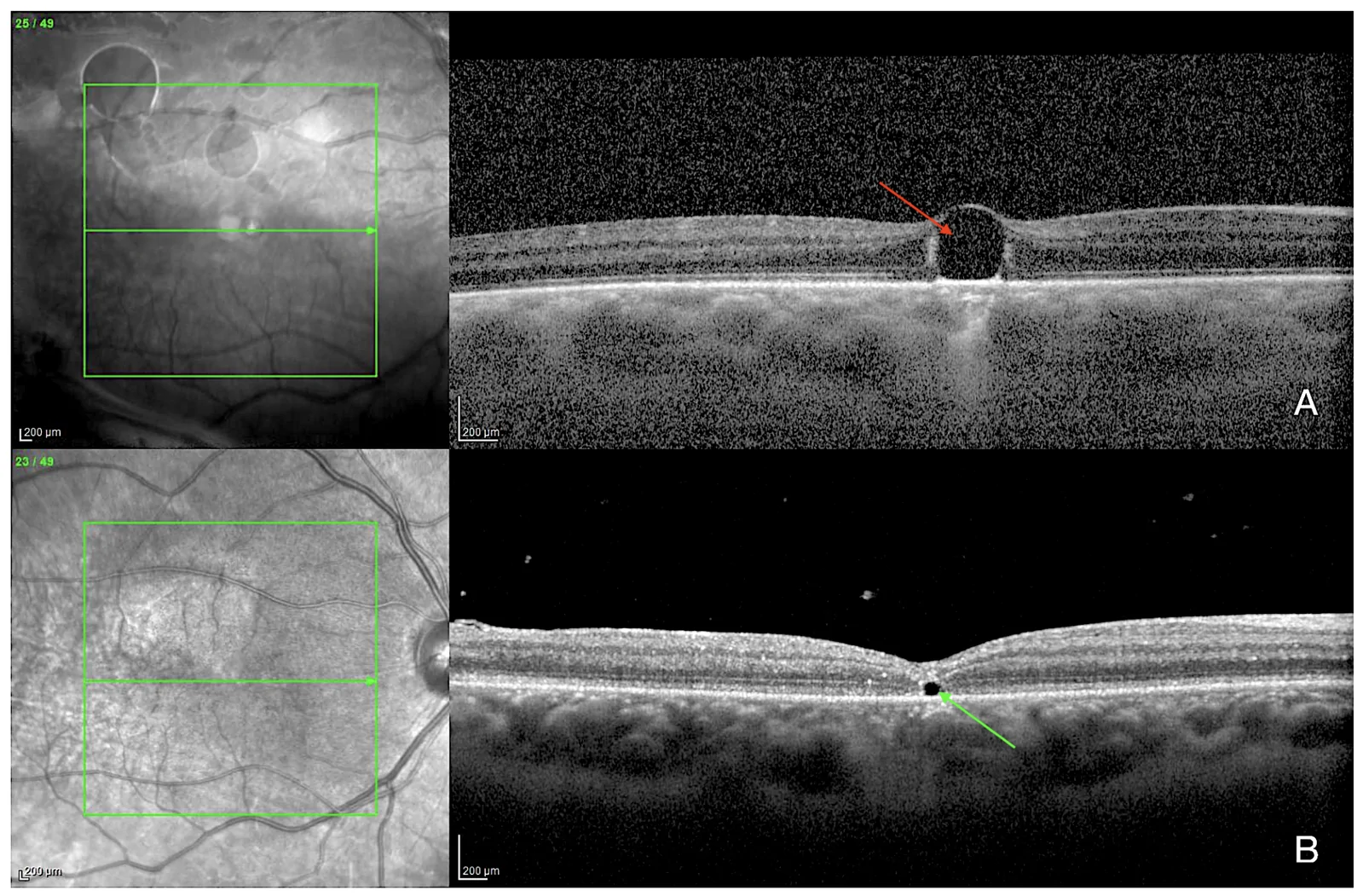
Case 2. (**A**) Preoperative OCT showing a subfoveal PFCL droplet (red arrow) with a maximum vertical height of 316 μm and a localized RPE defect. (**B**) OCT at the 4-week follow-up showing no residual PFCL, a focal outer retinal defect at the fovea (green arrow), and a reduction in the size of the RPE defect compared with the preoperative scan.

**Figure 5:**
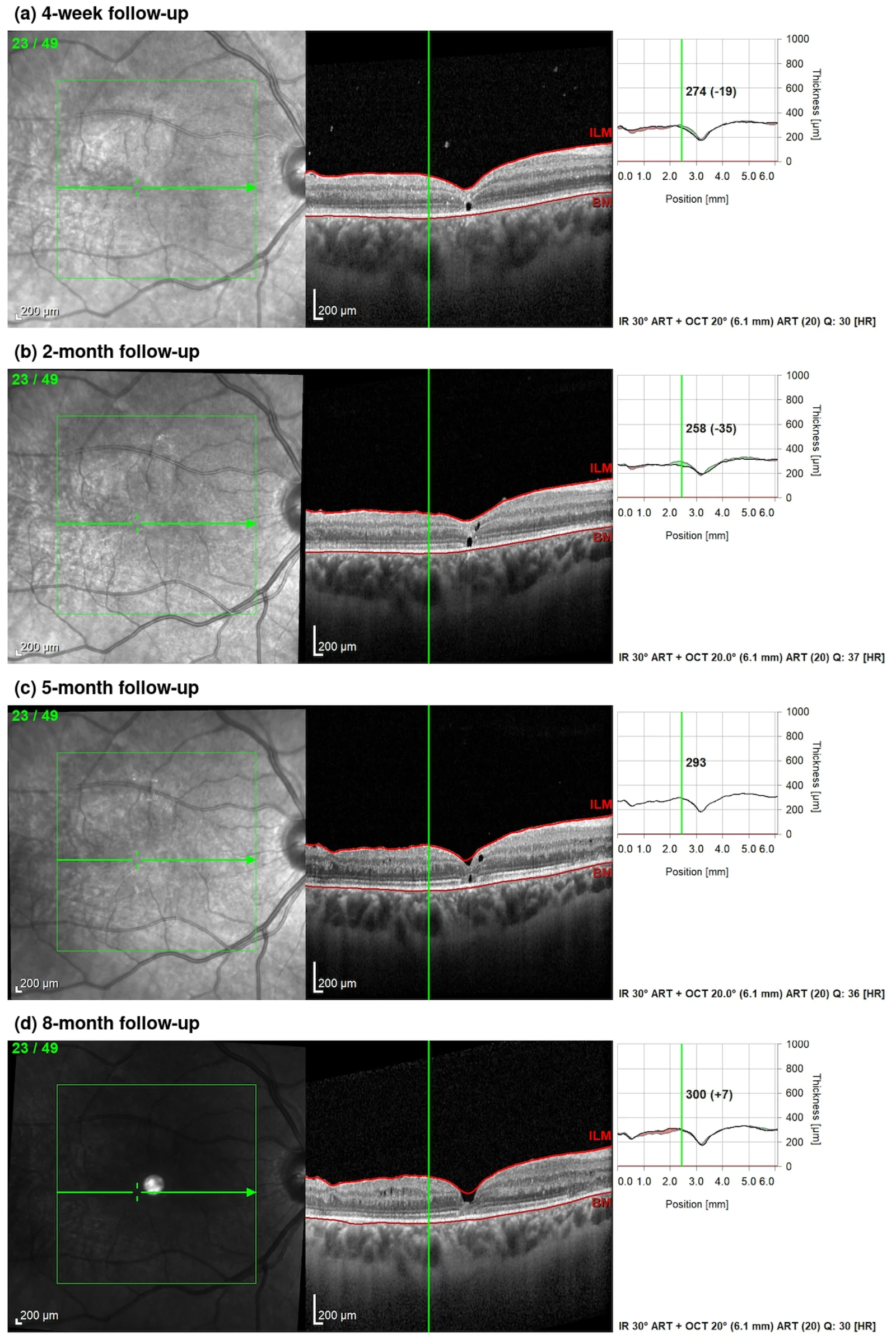
Case 2. Serial OCT scans obtained during follow-up showing progressive restoration of the outer retinal layers and foveal architecture.

**Figure 6:**
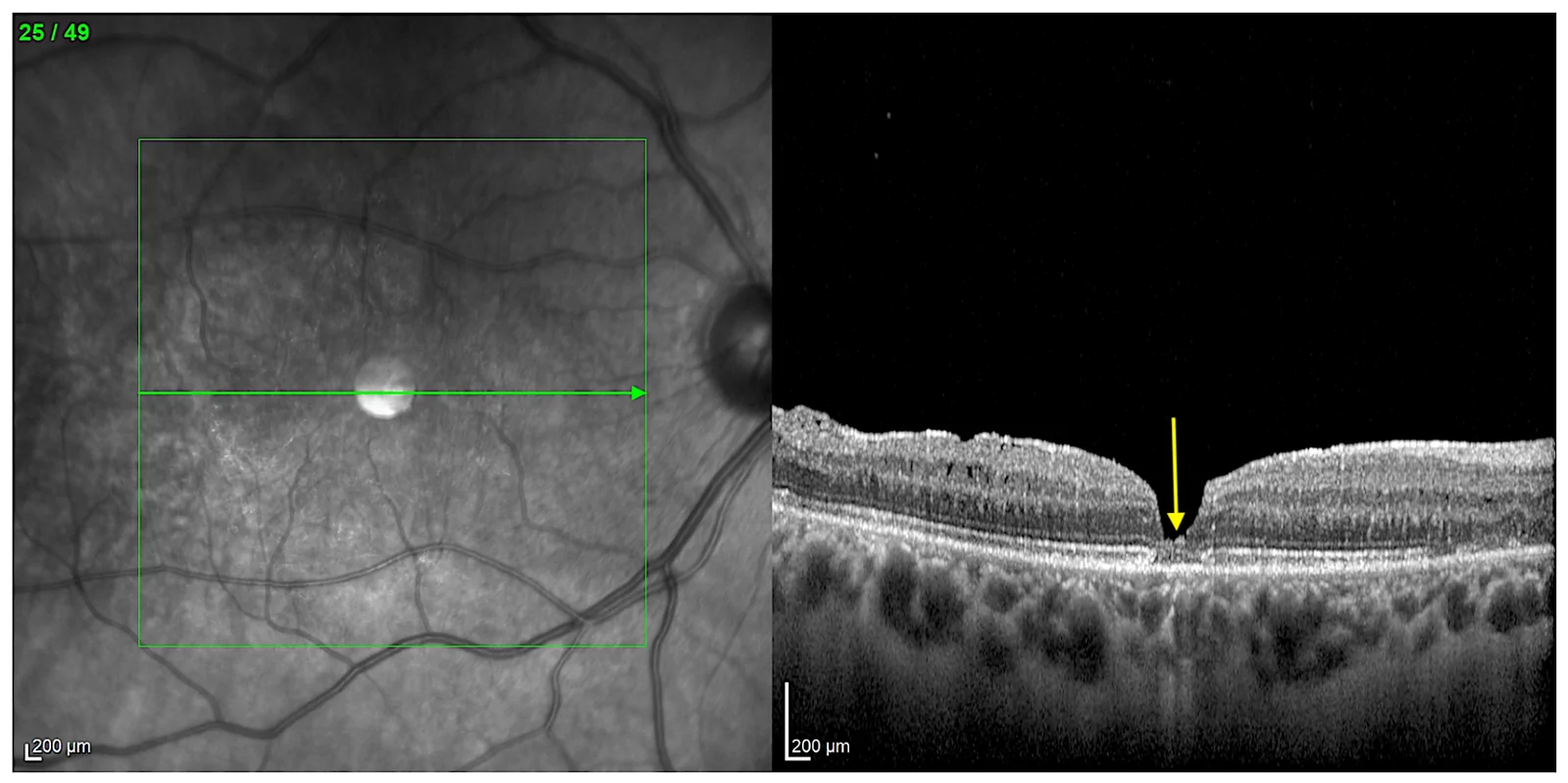
Case 2. Final OCT at the 8-month follow-up showing substantial restoration of the outer retinal layers and foveal contour at the previous PFCL location (yellow arrow), with residual focal photoreceptor abnormalities and a small area of inner retinal layer splitting.

**Figure 7:**
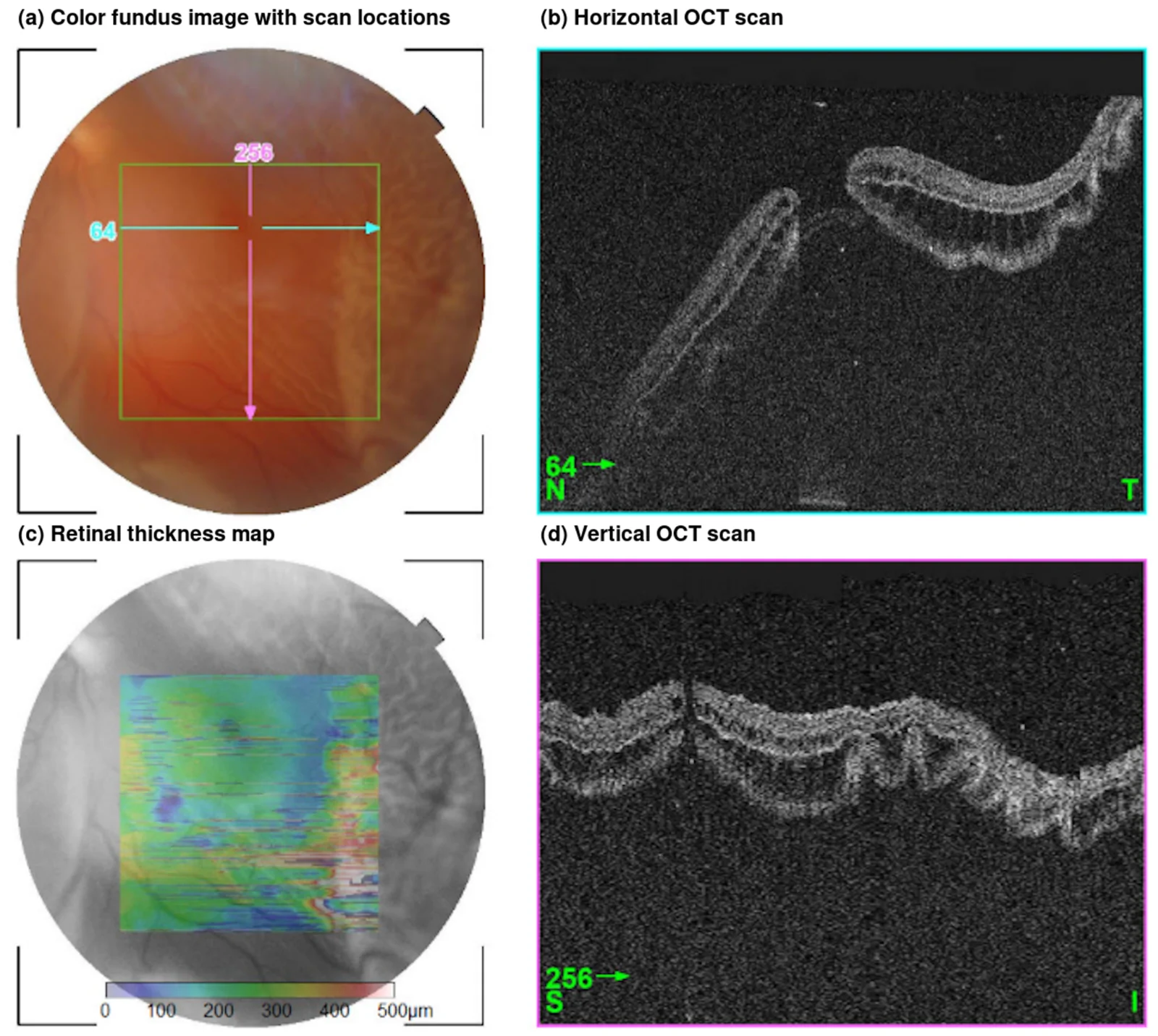
Case 3. OCT at presentation showing macular detachment with a full-thickness macular hole.

**Figure 8:**
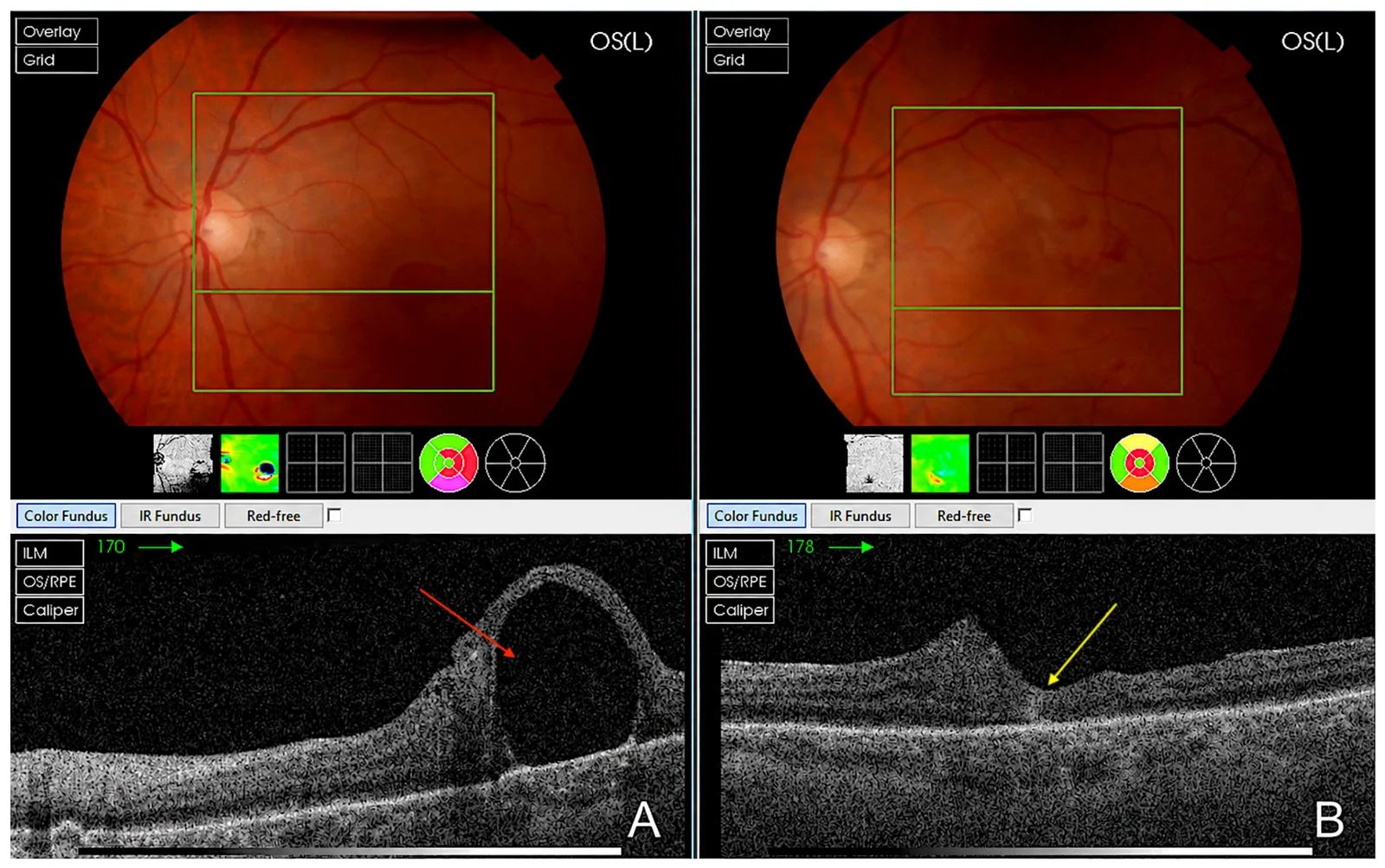
Case 3. (**A**) Preoperative OCT showing a parafoveal PFCL droplet (red arrow) with a maximum vertical height of 930 μm, deformation of the outer retinal layers, and focal RPE disruption. (**B**) OCT at the 1-week follow-up showing no residual PFCL and reapposition of the overlying retina, with focal retinal layer disorganization and localized thickening at the previous PFCL location (yellow arrow).

**Figure 9:**
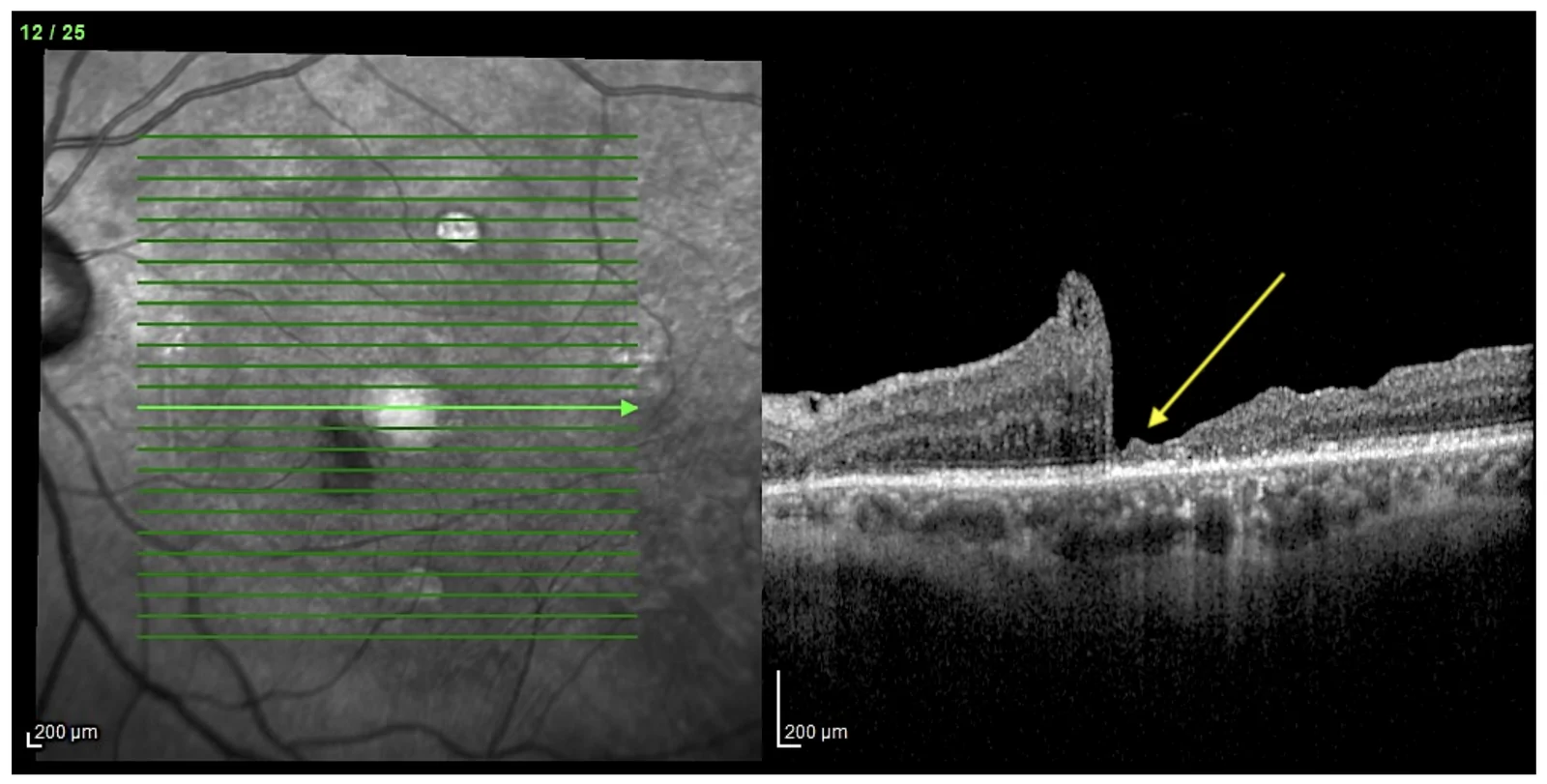
Case 3. Final OCT at the 33-month follow-up showing no residual PFCL and preserved overall retinal layer continuity, despite focal architectural disorganization, localized retinal thickening, and focal defects of the RPE and outer retinal layers at the previous PFCL location (yellow arrow).

**Table 1:** Clinical course and visual outcomes following subretinal PFCL removal.

Parameter	Case 1	Case 2	Case 3
Age/sex	54/M	35/M	50/M
PFCL location	Parafoveal	Subfoveal	Parafoveal
PFCL detection after primary surgery	33 days	31 days	22 days
PFCL removal after primary surgery	55 days	38 days	43 days
BCVA before PFCL removal (logMAR)	1.0	1.7	CF at 3 m (≈1.3)
Early postoperative BCVA (logMAR)	CF (≈1.9) at 10 days	HM (≈2.3) at 4 weeks	HM (≈2.3) on day 1; 1.4 at 1 week
Concomitant surgical procedures	None *	Phacoemulsification, BIL IOL implantation, silicone oil removal, endolaser photocoagulation	None
Final tamponade	Air	12% C_2_F_6_	Air
Final BCVA (logMAR)	0.4	0.05	1.0
Follow-up duration	9 months (280 days)	8 months (253 days)	33 months (1010 days)

Abbreviations: BCVA, best-corrected visual acuity; BIL, bag-in-the-lens; CF, counting fingers; HM, hand motion; IOL, intraocular lens; logMAR, logarithm of the minimum angle of resolution; PFCL, perfluorocarbon liquid. CF and HM values were converted to approximate logMAR equivalents for descriptive purposes and should not be considered equivalent to standardized chart-based measurements [12,13]. * In Case 1, iris repositioning was required on the first postoperative day due to iris capture.

## Data Availability

The data are not publicly available due to patient confidentiality and privacy restrictions.

## Supplementary Materials

The following supporting information can be downloaded at: https://www.mdpi.com/article/10.3390/jcm15218344/s1. Supplementary Video S1. Surgical video of Case 2 illustrating the ILM flap-assisted technique for subretinal PFCL removal. The video demonstrates the main surgical steps, including intraoperative OCT visualization of the retained PFCL droplet (00:00), creation of a temporally hinged ILM flap (00:03), stabilization of the flap with PFCL (00:10), and subretinal PFCL aspiration using a 39-gauge cannula (00:15). Subsequent intraoperative OCT (00:20) confirms complete removal of the retained subretinal PFCL. The video concludes with PFCL-assisted repositioning of the ILM flap (00:24).

## Abbreviations

The following abbreviations are used in this manuscript:

BCVA	Best-corrected visual acuity
BIL	Bag-in-the-lens
BSS	Balanced salt solution
CF	Counting fingers
ERM	Epiretinal membrane
HM	Hand motion
ICG	Indocyanine green
ILM	Internal limiting membrane
IOL	Intraocular lens
LASIK	Laser-assisted in situ keratomileusis
logMAR	Logarithm of the minimum angle of resolution
OCT	Optical coherence tomography
PFCL	Perfluorocarbon liquid
PVR	Proliferative vitreoretinopathy
RPE	Retinal pigment epithelium
